# Prediction of clinical response to neoadjuvant therapy in advanced breast cancer by baseline B-mode ultrasound, shear-wave elastography, and pathological information

**DOI:** 10.3389/fonc.2023.1096571

**Published:** 2023-05-09

**Authors:** Siyu Wang, Wen Wen, Haina Zhao, Jingyan Liu, Xue Wan, Zihan Lan, Yulan Peng

**Affiliations:** Department of Medical Ultrasound, West China Hospital, Sichuan University, Chengdu, Sichuan, China

**Keywords:** advanced breast cancer, B-mode ultrasound, shear-wave elastography, neoadjuvant therapy, clinical response prediction

## Abstract

**Background:**

Neoadjuvant therapy (NAT) is the preferred treatment for advanced breast cancer nowadays. The early prediction of its responses is important for personalized treatment. This study aimed at using baseline shear wave elastography (SWE) ultrasound combined with clinical and pathological information to predict the clinical response to therapy in advanced breast cancer.

**Methods:**

This retrospective study included 217 patients with advanced breast cancer who were treated in West China Hospital of Sichuan University from April 2020 to June 2022. The features of ultrasonic images were collected according to the Breast imaging reporting and data system (BI-RADS), and the stiffness value was measured at the same time. The changes were measured according to the Response evaluation criteria in solid tumors (RECIST1.1) by MRI and clinical situation. The relevant indicators of clinical response were obtained through univariate analysis and incorporated into a logistic regression analysis to establish the prediction model. The receiver operating characteristic (ROC) curve was used to evaluate the performance of the prediction models.

**Results:**

All patients were divided into a test set and a validation set in a 7:3 ratio. A total of 152 patients in the test set, with 41 patients (27.00%) in the non-responders group and 111 patients (73.00%) in the responders group, were finally included in this study. Among all unitary and combined mode models, the Pathology + B-mode + SWE model performed best, with the highest AUC of 0.808 (accuracy 72.37%, sensitivity 68.47%, specificity 82.93%, P<0.001). HER2+, Skin invasion, Post mammary space invasion, Myometrial invasion and Emax were the factors with a significant predictive value (P<0.05). 65 patients were used as an external validation set. There was no statistical difference in ROC between the test set and the validation set (P>0.05).

**Conclusion:**

As the non-invasive imaging biomarkers, baseline SWE ultrasound combined with clinical and pathological information can be used to predict the clinical response to therapy in advanced breast cancer.

## 1 Introduction

Breast cancer has now surpassed lung cancer as the world’s largest cancer, ranking first globally and fourth in China in the spectrum of cancer deaths in women (1, 2). Advanced breast cancer (ABC), including locally advanced breast cancer (LABC), and metastatic breast cancer, which cannot undergo radical surgery at present, are related to the high incidence of metastasis and poor prognosis (3, 4). Therefore, the main goal of its treatment is to delay the progress of the disease, prolong the survival time, and improve the quality of life of patients. Neoadjuvant therapy (NAT) and salvage therapy for M1 stage breast cancer, instead of surgical resection at diagnosis, are recommended as the preferred treatment to ABC to provide more surgical opportunities and improve the survival rate according to the National Comprehensive Cancer Network (NCCN) guidelines (5). Therefore, the accurate evaluation of the curative effect is of particular significance. At present, the evaluation of clinical response is mainly carried out through pathological and clinical methods, that is, preoperative Response Evaluation Criteria in Solid Tumors (RECIST1.1) (6) and postoperative Miller–Payne (MP) Grading Criteria classification (7). In clinical application, the timing of surgery and intraoperative tumor resection volume are based on clinical response (8), which should be predicted as early as possible and focused on during treatment.

Studies have proven that breast images can provide tumor biology behavior from multiple aspects but focus more on the changes during disease treatment. In the initial stage, the tumor baseline images can better reflect the original characteristics of the tumor. Ultrasonography is a low-cost imaging modality that increases cancer sensitivity and detection rates in dense breast populations. China has a relatively higher proportion of dense breast lesions than other countries (9), which explains the popularity and importance of ultrasound for Chinese breast screening. Moreover, with the continuous innovation of technology, multimodal ultrasound technology is more and more advocated because of its multiparameter and all-around evaluation ability. Shear wave elastography (SWE), a new technology in clinical applications in recent years, can provide quantitative information by measuring the stiffness of breast masses (10, 11). Adding quantitative SWE parameters to the BI-RADS feature in breast masses has been applied in clinical use to differentiate benign and malignant tumors, improving the specificity of breast US mass assessment without loss of sensitivity, especially in characterizing a complex lesion (12, 13). Furthermore, the deep learning model, convolutional neural network (CNN) based on SWE parameters, greatly improves the accuracy and reliability of computer-aided diagnosis, which can be used for the detection and management of breast cancer (14, 15). Studies have shown that tumors with high stiffness are more likely to be associated with metastasis and poor prognosis (16, 17). The decrease in tumor stiffness during treatment is related to the curative effect (8). Nevertheless, the establishment of relevant models still needs more experiments, especially the application at the early stage.

This study aims to add imaging information to the traditional clinical and pathological indicators and predict the clinical response to therapy for advanced breast cancer through the tumor baseline situation, moving the prediction period forward to provide critical information for clinical treatment.

## 2 Materials and method

### 2.1 Patient characteristic

This retrospective, single-center study was conducted by the Declaration of Helsinki and approved by the West China Hospital of Sichuan University Biomedical Research Ethics Committee. All participants provided informed consent for inclusion before participation in the study.

**Figure 1:**
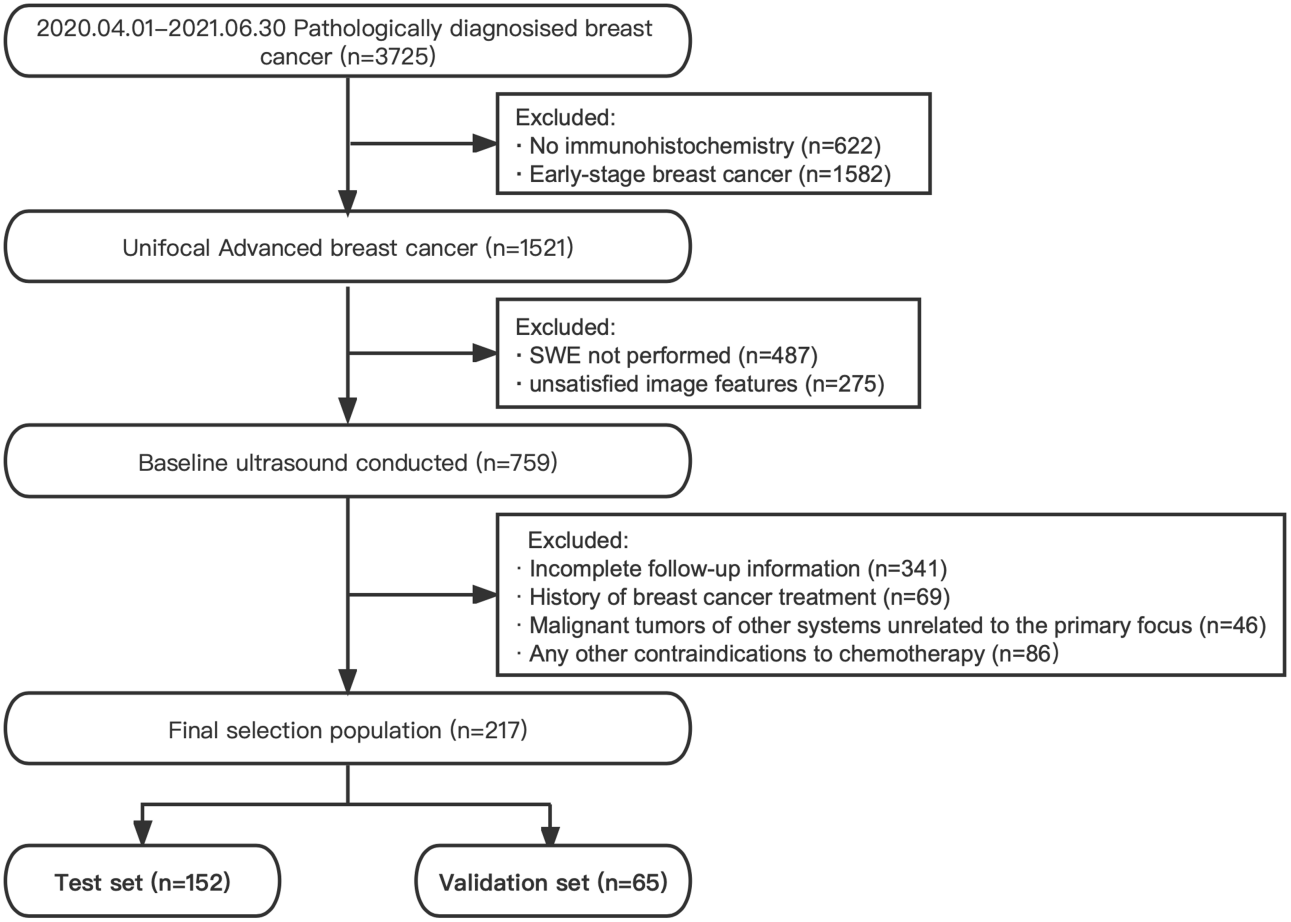
Flow diagram of the subject selection.

The subjects were collected at West China Hospital of Sichuan University from April 2020 to June 2022. The inclusion criteria were as follows: (I) unifocal advanced breast cancer (T0~2N2M0, T3N0~2M0, T0~4N3M0, and T0~4N0~3M1) (18), (II) B-mode and SWE ultrasound examinations performed within 30 days before intervention, and (III) followed up for clinical response evaluation. The exclusion criteria were as follows: (I) previous treatment history, (II) primary malignancy in other organs, (III) any contraindications to therapeutic drugs, and (IV) pregnant women. All participants received standard cycle treatment according to standard protocols mentioned in the NCCN guidelines (5). The flow diagram of subject selection is shown in **Figure 1**.

### 2.2 Pathology information

Immunohistochemistry (IHC) and fluorescence *in situ* hybridization (FISH) tests were conducted for receptor expression estimate. Estrogen receptor (ER) and progesterone receptor (PR) were recorded in the form of positive (+) or negative (−) and percentage (%) expression according to the American Society of Clinical Oncology/College of American Pathologists (ASCO/CAP) guideline (19). HER2+ was defined as HER2 3+ or FISH+, and the others were defined as HER2− (5). Ki-67 was directly reported as the percentage of positively stained nuclei. All tumors were divided into four IHC subtypes according to the St. Gallen criteria (20).

### 2.3 Ultrasound examinations

All patients underwent B-mode and SWE ultrasound examinations within 30 days before intervention (baseline), using Siemens OXANA2 ABVS ultrasonic device (Siemens Healthineers, Munich, Germany) equipped with 18L6 high frequency (15 MHz) and 9L4 linear-array (8 MHz) transducers. Radiologists have more than 5 years of breast diagnosis experience and, as one of the multi-center units, have unified requirements and training on operation technology according to the Chinese Guidelines and Recommendations on the Clinical Use of Ultrasound Elastography (21).

First, to obtain the best B-mode ultrasound image, the major axis plane and plane vertical to it were acquired for each mass for measuring tumor size. Three diameters were recorded, and volume was calculated according to them. Images of each breast mass were interpreted according to ACR BI-RADS Atlas Fifth Edition (22) and documented the ultrasound imaging features, including maximum diameter (dmax), volume, orientation (parallel, not parallel), margin (regular, indistinct, angular, micro-lobulated, and spiculated), calcifications (absence or presence of suspicious calcifications), echo pattern (hypoechoic, isoechoic, and heterogeneous), posterior features (no posterior features, enhancement, shadowing, and combined pattern), peripheral tissue involvement (architectural distortion, duct changes, skin thickening, and skin edema), and invasion layers (skin, subcutaneous fat, gland, posterior mammary space, and muscle). The blood supply of the tumor was evaluated by Adler grades, and lymph node involvement was evaluated at the same time.

**Figure 2:**
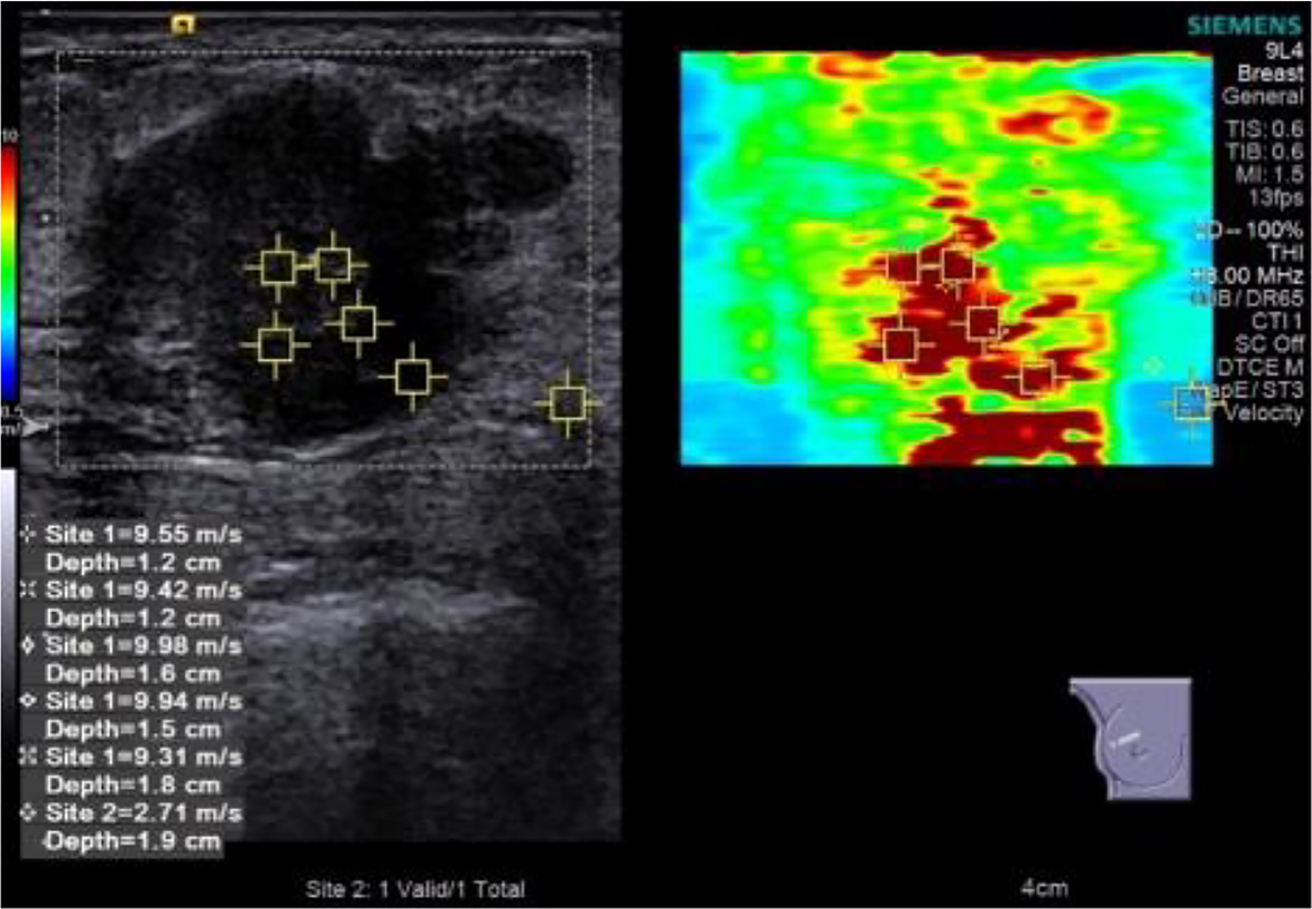
Elastic value acquisition: five points in the tumor’s hard (red) area and one point in the peripheral normal adipose tissue.

Then, the depth, focus, gain, local amplification, and other conditions were optimized and switched to SWE mode. The range scale is uniformly selected at 10 m/s. The patient was asked to hold his breath to reduce the impact of breathing movement on the image. After holding the probe until the elastic image remains stable for several seconds, the image was collected and played back, and the image was taken with the best color signal filling for quantitative measurement. The square region of interest (ROI) used for SWE acquisition was adjusted to include the entire mass and surrounding normal tissue observed in B-mode, excluding the skin and chest wall. In ROI, the default stiffness range was from blue to red (soft to hard). *The examiner selected five points in the hardest area for elastic value collection (Site1)* and one point in peripheral normal adipose tissue (Site2) (**Figure 2**). The system calculated the maximum lesion stiffness (Emax), minimum (Emin), median (Emedian), mean (Emean), and standard deviation (Estd) automatically. Given that the system displays “High” when lesions velocity is higher than 10 m/s, these cases were set equivalent to the maximum value of 10 m/s for analysis.

### 2.4 Outcome

After completing the standard adjuvant treatment process, the oncologist conducted a comprehensive clinical and image evaluation (by MRI) on the curative effect according to RECIST1.1 guidelines (6) as follows:

- Progressive disease (PD): (I) at least a 20% increase in the sum of diameters of target lesions and demonstrating an absolute increase of at least 5 mm, (II) the appearance of new malignant lesions, and (III) a lesion identified on a follow-up study in an anatomical location that was not scanned at baseline.
- Stable disease (SD): (I) the smallest sum diameters of target lesion, neither sufficient shrinkage to qualify for PR nor sufficient increase to qualify for PD and (II) non-target lesions not all evaluated.
- Partial response (PR): (I) at least a 30% decrease in the sum of diameters of target lesions, taking as reference the baseline sum diameters and (II) persistence of one or more non-target lesion(s) and/or maintenance of tumor marker level above the normal limits.
- Complete response (CR): (I) disappearance of all target lesions, (II) any pathological lymph nodes (whether target or non-target) reduction in short axis to<10 mm, and (III) disappearance of all non-target lesions and normalization of tumor marker level.

We defined PD and SD as non-responders while PR and CR as responders.

### 2.5 Statistical analysis

Our final selection population was split into a test set for model development and a validation set in a 7:3 ratio. Univariate analyses were performed in the test set using Student’s t-test, Mann–Whitney U-test, Pearson’s chi-squared test, or Fisher’s exact test to examine the factors associated with tumor response. For the multivariable analysis, we selected those covariates with p-values<.2 in the univariate analysis. The odd ratio (OR) and 95% confidence interval [CI] value of significant predictors were determined by the single or combined regression model. A receiver operating characteristic (ROC) curve and the area under the curve (AUC) were generated to assess the discriminative ability of the prediction model. Sensitivity and specificity were calculated using the best cutoff score for the clinical prediction rule and the Youden index for the ROC. In the validation set, the application effectiveness of the combined model was evaluated with AUC and Z-test. In all the analyses, p<0.05 were considered significant. Data were analyzed using SPSS v26.0 (SPSS, Inc., IMB Company Chicago, IL, USA).

## 3 Result

### 3.1 Clinical and pathological indicators

A total of 152 patients were finally included in the test set, with an average age of 47.98 ± 9.36 years at diagnosis. The initial clinical stages were T stages 1, 2, 3, and 4 (4.61%, 30.92%, 19.74%, and 44.74%); N stages 0, 1, 2, and 3 (3.29%, 22.37%, 36.84%, and 37.50%); and M stages 0 and 1 (84.21% and 15.79%). Invasive ductal carcinoma accounted for 81.58%, and other histological types accounted for 18.42%. According to the outcome indicators, 41 patients (27.00%) were divided into the non-responders group and 111 patients (73.00%) into the responders group. Age, T, N, and M stage, and histological type were not significantly correlated with the clinical response after NAT (p=0.831, 0.580, 0.905, 0.444, and 0.464, respectively (**Supplementary Appendix Table A1**).

It showed that HER2+ was a significantly predictive indicator of clinical response, with 50.45% in responders group and 17.07% in non-responders group (p<0.001). There were no differences in the expression of ER%, ER+/−, PR%, PR+/−, Ki-67%, and IHC subtypes between responders and non-responders groups (p>0.05). In the pathology regression model, the factor with a significant predictive value was HER2+ (OR, 4.945; 95% CI, 2.022–12.098; p<0.001). The prediction performance of one modality (Pathology) is listed in **Table 1**, with an AUC of 0.667 (p=0.002), an accuracy of 59.21%, a sensitivity of 50.45%, and a specificity of 82.93%.

**Table 1:** AUC-ROC, sensitivity and specificity of one modality, two modalities, and three modalities and validation set.

	AUC-ROC	95% CI	Accuracy (%)	Sensitivity (%)	Specificity (%)	p-value
1 modality						
Pathology	0.667	0.575-0.759	59.21	50.45	82.93	0.002
B-mode	0.712	0.623-0.801	58.55	48.65	85.37	<0.001
SWE	0.585	0.493-0.676	57.24	47.75	82.93	0.110
2 modalities						
Pathology + B-mode	0.796	0.721-0.870	68.42	62.16	85.37	<0.001
Pathology + SWE	0.712	0622-0.801	73.68	74.77	70.73	<0.001
B-mode + SWE	0.674	0.586-0.763	63.16	57.66	78.05	0.001
3 modalities						
Pathology + B-mode + SWE	0.808	0.586-0.763	72.37	68.47	82.93	<0.001
Validation Set	0.755	0.655-0.870	72.31	70.00	80.00	<0.001

AUCs, areas under the curve; ROC, receiver operating characteristic curve; CI, confidence interval; SWE, shear wave elastrography.

p< 0.05, the difference is statistically significant.

### 3.2 B-Mode ultrasound features

For size, we measured for responders and non-responders group; dmax was mean of 42.63 ± 21.64 *vs*. 50.88 ± 25.13 mm (p=0.060), and volume was mean of 37,894.73 ± 57,625.57 *vs*. 71,956.78 ± 112,500.60 mm^3^ (p=0.105). For features, mass without micro-lobulated margin (43.24% [48 of 111] *vs*. 21.95% [9 of 41]; p=0.016), mass without spiculated margin (80.18% [89 of 111] *vs*. 60.97% [25 of 41]; p=0.015), mass without skin thickening (79.28% [88 of 111] *vs*. 63.41% [26 of 41]; p=0.045), mass without skin invasion (81.98% [91 of 111] *vs*. 63.41% [26 of 41]; p=0.016), and mass without posterior mammary space invasion (19.81% [22 of 111] *vs*. 4.88% [2 of 41]; p=0.025) were more frequently observed in the responders group than in the non-responders group. Furthermore, 1.80% (2 of 111) of BI-RADS 4b, 16.22% (18 of 111) of BI-RADS 4c, 81.98% (91 of 111) of BI-RADS 5 obtained the response also with statistical differences (p=0.034). However, we found no significant differences in the proportions of regular margin (0 of 152), angular margin (p=0.291), parallel (p=0.262), calcifications (p=0.517), posterior enhancement (p=0.144), posterior shadowing (p=0.381), posterior combined pattern (p=0.155), duct change (p=1.000), skin edema (p=0.053), subcutaneous fat invasion (p=0.460), myometrial invasion (p= 0.053), nipple invasion (p=1.000), lymph nodes (p=0.294), and Adler grads (p=0.802) between the responders group and non-responders group (**Supplementary Appendix Table A2**).

In the B-mode model, volume (OR, 1.000; 95% CI, 1.000–1.000; p=0.008), spiculated margin (OR, 0.431; 95% CI, 0.191–0.976; p=0.043), and myometrial invasion (OR, 0.353; 95% CI, 0.136–0.914; p=0.032) were the factors with a significant predictive value. The AUC under ROC was 0.712, with an accuracy of 58.55%, a sensitivity of 48.65%, and a specificity of 85.37% (p<0.001) (**Table 1**).

### 3.3 SWE parameters

**Table 2** summarizes the relationship between SWE parameters and tumor response. The comparison of Emin and Emean was statistically significant with 8.31 ± 1.62 *vs*. 7.47 ± 2.11m/s (p*=*0.043) and 8.97 ± 1.29 *vs*. 8.13 ± 1.93 m/s (p*=*0.046). No significant difference was found in Emax (9.54 ± 1.15 *vs*. 8.74 ± 1.80 m/s, p*=*0.109), Emedian (8.98 ± 1.34 *vs*. 8.17 ± 2.03 m/s, p=0.098), and Estd (0.52 ± 0.04 *vs*. 0.58 ± 0.46, p=0.602) between the two groups. The AUC of ROC made by regression logistic (SWE model) was 0.585, with an accuracy of 57.24%, a sensitivity of 47.75%, and a specificity of 82.93% (p=0.110) (**Table 1**).

**Table 2:** Summary of the SWE parameters for the 152 patients.

Parameters	Mean ± SD		Minimum		Maximum		Median		p-value
	Non-responders (n=41)	Responders (n=111)	Non-responders (n=41)	Responders (n=111)	Non-responders (n=41)	Responders (n=111)	Non-responders (n=41)	Responders (n=111)	
Emax	9.54 ± 1.15	8.74 ± 1.80	3.07	3.28	10.00	10.00	9.87	9.76	*0.109*
Emin	8.31 ± 1.62	7.47 ± 2.11	1.94	2.77	10.00	10.00	8.67	8.05	*0.043*
Emedian	8.98 ± 1.34	8.17 ± 2.03	2.68	2.85	10.00	10.00	9.41	9.22	*0.098*
Emean	8.97 ± 1.29	8.13 ± 1.93	2.63	2.97	10.00	10.00	9.31	8.92	*0.046*
Estd	0.52 ± 0.40	0.58 ± 0.46	0.00	0.00	1.59	2.47	0.46	0.48	0.602

Data are mean ± standard deviation. p-values for difference were determined by Mann–Whitney U test; p<0.05, the difference is statistically significant.

### 3.4 Predictive models development

**Figure 3:**
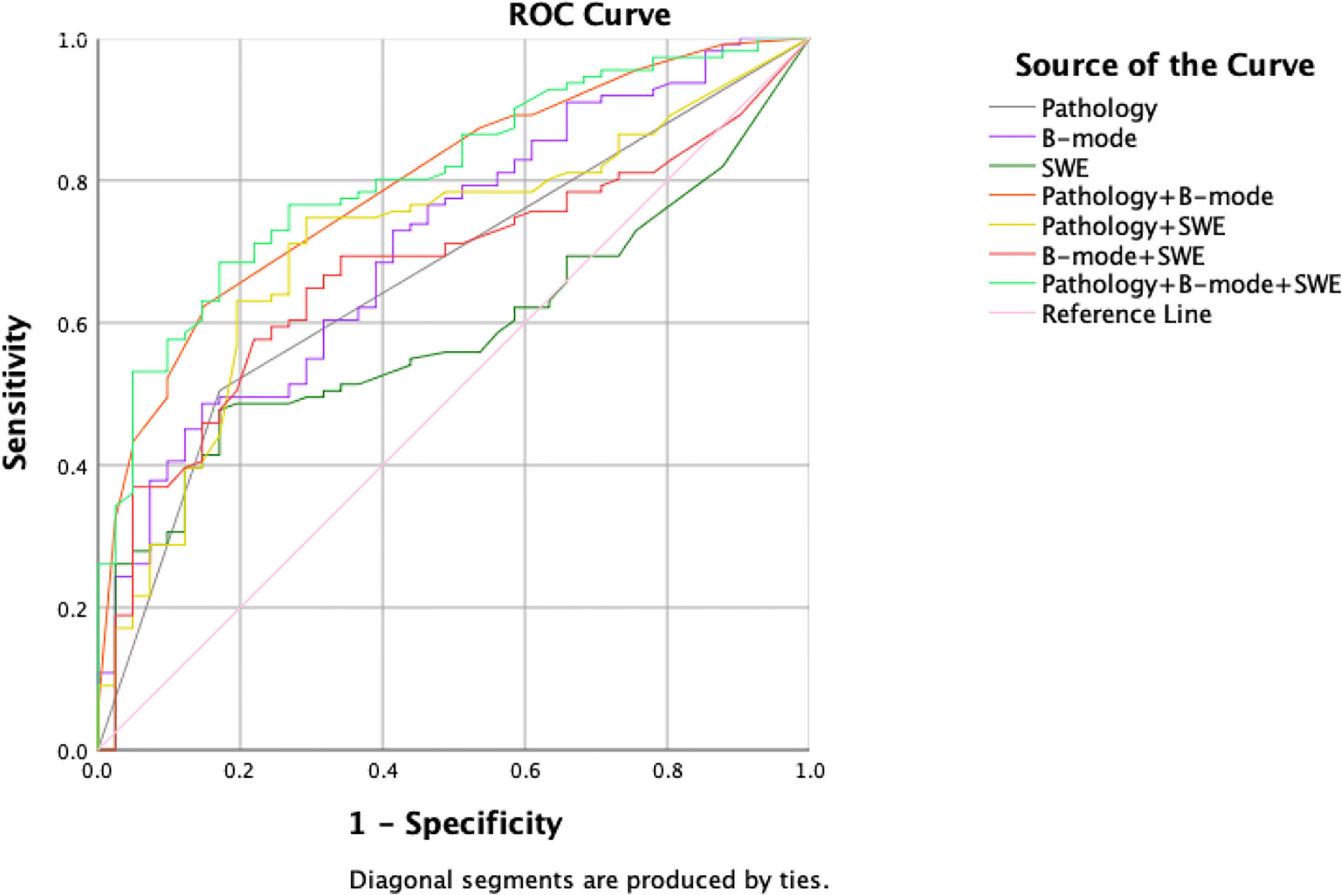
ROC curve summary of one-, two-, and three-modalities models.

All multivariate regression models are summarized in **Table 1**. Among the one-, two-, and three-modalities combined prediction models, the Pathology + B-mode + SWE model performed best, with the highest AUC of 0.808 (95% CI, 0.737–0.879; accuracy, 72.37%; sensitivity, 68.47%; specificity, 82.93%, p<0.001). The second is the Pathology + B-mode model with an AUC of 0.796 (95% CI, 0.721–0.870; accuracy, 68.42%; sensitivity, 62.16%; specificity, 85.37%; p<0.001). B-mode and Pathology + SWE models show the same AUC of 0.712, and the remaining prediction models were lower than the above, with an AUC< 0.700 (**Figure 3**).

In the optimal prediction model (three modalities), HER2+(OR, 8.541; 95% CI, 2.966–24.595; p<0.001), skin invasion (OR, 0.236; 95% CI, 0.085–0.654; p*=*0.006), post-mammary space invasion (OR, 0.178; 95% CI, 0.036–0.886; p=0.035), myometrial invasion (OR, 0.284; 95% CI, 0.096–0.842; p=0.023), and Emax (OR, 0.672; 95% CI, 0.471–0.959; p=0.028) were the factors with a significant predictive value (**Table 3**).

**Table 3:** Independent influencing factors in three modalities (Pathology + B-mode US + SWE) model.

Factors	B	OR	95% CI	p-values
HER2+	2.145	8.541	2.966-24.595	<0.001
Skin invasion	-1.443	0.236	0.085-0.654	0.006
Post mammary space invasion	-1.725	0.178	0.036-0.886	0.035
Myometrial invasion	-1.259	0.284	0.096-0.842	0.023
Emax	-0.397	0.672	0.471-0.959	0.028

OR, odds ratio; HER2+, human epidermal growth factor receptor-2 positive; Emax, maximum lesion stiffness.

p<0.05, the difference is statistically significant.

Based on the data in **Table 3**, we established the following logistic model:


$$p=1/1+\text{Exp}\sum \begin{array}{l} {}[6.123+2.145\times (if\;HER2+)-1.443\times (\text{if skin invasion on US})-1.725\times (if\;post\;-\;\\ mammary\;space\;invasion\;on\;US)-1.259\times (if\;myometrial\;ivasion\;on\;US)-0.397\times (Emax)] \end{array}$$

### 3.5 Validation of predictive model

The calculated p-value was compared with the probability value of the cutoff point of the final combined model. Greater than means a response, and less than means no response. The distribution of all the variables were statistically not different between test and validation sets (all, p>0.05) (**Supplementary Appendix Tables B1**-**3**). The outcomes are grouped according to the cutoff value in the validation set and then validated. There were 35 true positives, 3 false positives and 15 false negatives, 12 true negatives. The AUC of the validation set was 0.775 (95% CI, 0.655–0.870, p<0.001) (**Table 1**). Compared with the three-modalities model, the AUC was 0.775 *vs*. 0.808, the accuracy was 72.31% *vs*. 72.37%, the sensitivity was 70.00% *vs*. 68.47%, and the specificity was 80.00% *vs*. 82.93%. After Z-test, there was no statistical difference in ROC between the test set and the validation set (p>0.05).

## 4 Discussion

In this study, the clinical and ultrasonic characteristics and SWE parameters of 152 patients with advanced breast cancer were analyzed to obtain efficacy predictors and establish prediction models, which were well-validated in 65 validation sets. The research results show that histological characteristics, baseline B-mode characteristics, and SWE parameters are all related to the clinical response of adjuvant therapy. The combined prediction model of the three can improve the prediction ability to a certain extent.

We observed that there is no statistical significance in the clinical TNM stage. At the same time, it is not consistent with the results of recent studies, which pointed out that cT1/cT2 can be associated with a good prognosis (23), and high lymph node burden (N stage) will indicate adverse prognosis (24). The main reason may be the generally large tumor diameter and volume of advanced breast cancer and the high proportion of T4 (44.74%), and 96.05% of them are accompanied by lymph node metastasis, resulting in no significant difference in statistical analysis.

In our study, the expression of HER2 is significantly correlated with tumor response, which is in agreement with the subjects and results of a retrospective study by Zheng et al. They confirmed that the response of adjuvant therapy is equivalent to that of NAT in patients with HER2+ and emphasized that patients with cT3/4 or those with positive clinical nodal status were more likely to benefit from NAT (25). Because of the overexpression of the oncogene ERBB2, HER2+ promotes the growth of cancer cells, which results in positive progress and a worse prognosis (26). However, as a therapeutic target, HER2+ BC has been proven to be more sensitive to targeted therapy than other IHC subtypes (27, 28). In each prediction model, HER2+ showed a great contribution that reemphasized its importance. However, the correlation between other biomarkers or IHC subtypes and clinical response is not found in our study, which differs from the report of ER+ and Ki-67% as diagnostic predictors proposed by some previous studies (29–32). It may be the deviation caused by sample size and different proportions of IHC subtypes, and the above studies are mostly focused on a certain subtype.

The association between baseline US images and adjuvant treatment outcomes was also demonstrated. Some studies have shown that tumors with larger dmax or metabolic volume are more likely to have a poor response (33, 34); dmax and volume of lesions in non-responders group were also relatively larger in this study, although p>0.05. As discussed above, the recognized relationship between tumor size and prognosis may have been lost for relatively large tumors in the late stage. On the other hand, the image measurement error is large, and the vertical aspect ratio judgment may be inaccurate to a certain extent.

Studies of baseline image features of breast cancer at mammography and MRI have demonstrated that the tumor response is more likely in well-defined, oval or round lesions than in diffuse or irregular ones (33, 35). As we concluded, micro-lobulated and spiculated margins were negatively correlated with the response, which is also one of the common imaging features of all malignant tumors but may be more significant in advanced breast cancer. Additionally, skin, post-mammary space, and myometrial invasion were factors negatively correlated with treatment response in our models. Breast cancer generally occurs in the glandular layer, invading surrounding layers with its invasion and growth, which can be distinguished on ultrasonic images. Our results suggest that the tumor longitudinal, instead of the overall size for advanced breast cancer, is a dependently predictive prognostic factor. Results also showed that patients with non-skin thickening achieved more treatment response, which is proven to be tumor involving the skin, resulting in lymphatic and venous obstruction, massive invasion of subdermal connective tissue, and systemic metastasis (36). Evans and Wen reported that skin thickening (>2.5 mm) revealed by ultrasound imaging was independently related to lymph node load, and the 6-year metastasis-free survival (MFS) of women with skin thickening was worse (p=0.032, 6-year MFS 52% *vs*. 68%) (37, 38). Not parallel to the skin, calcifications and posterior features described by ultrasound were usually related to malignant tumors in histopathology (39, 40), which are not in this study. Such differences may indicate that in advanced breast cancer, these image features were common or difficult to distinguish due to fusion, resulting in decreased sensitivity of the prompt and not providing better prognostic information.

Emean and Emin were significantly correlated with NAT response in this study. Because all the elastic values come from the hard areas of the tumor, they reflect the elastic characteristics of the tumor to a greater extent. We can find no significant difference in tumor homogeneity between the two groups before treatment. However, there was a trend for the averaged stiffness in the responders group to be lower among all parameters, as a high SWE value is generally related to adverse prognosis because of the increase in extracellular matrix components of malignant tumors, the invasion of cancer cells into tissues, or the fibroproliferative reaction (41, 42). A meta-analysis reported that SWE-combined AUC of the NAC response was 0.82 (sensitivity, 79%; specificity, 81%) (43). Although our SWE model cannot independently predict tumor response (p>0.05), great predictive value has been shown in Emax for the combined model, which is consistent with Son that high Emean and Emax values were associated with invasive tumor size, high histological grade, and positive lymphatic vascular invasion(p<0.05), and could predict poor prognosis (44).

Wang summarized in a review that the combined application of various commonly used ultrasound technologies can well predict the response of NAT, with an AUC of 0.71–0.92 (45), and the addition of tumor clinicopathological information will improve the ability of the prediction model to a certain extent (30, 32). Different from previous studies on pathological response, we established the prediction model focused on clinical response, and the combined model shows the best prediction ability with an AUC of 0.808. The factors contributing to the model (HER2+, skin invasion, post-mammary space invasion, myometrial invasion, and Emax) were also highly consistent with the results of univariate analysis, suggesting that particular attention should be paid to these factors. Although in the combined model, compared with pathology and B mode, the addition of SWE does not significantly improve the AUC of the model. This may be attributed to the following reasons. First of all, the research proved that the diagnosis efficiency of SWE alone is lower than that of B-mode (46, 47), which is consistent with the model AUC obtained by our single SWE mode, indicating that the use of SWE needs to be based on conventional US, with additional reference information, rather than being used alone. In addition, SWE is more accurate for small tumors (48) and has limited ability to assess deep lesions (45). However, it still slightly improved the AUC of our prediction model, so the application of SWE in advanced breast cancer is the potential to some extent. It is a supplement to the information in different dimensions of conventional ultrasound while improving the accuracy and sensitivity of the model, although the specificity is slightly reduced, which can give more clinical indications to patients with poor prognoses to pay attention to them. In the future, more samples and groups will be needed for detailed analysis.

Our study has some limitations: (I) this study was performed in a single center, lacking regional representation; (II) the number of patients in the study is modest, so differences in performance according to immunophenotype have not been assessed; and (III) it is a retrospective study, but the ultrasonic examination method used in this study refers to a multicenter study of our research group (49) with specified unified standards.

## 5 Conclusions

Our study suggests that in patients with advanced breast cancer treated with NAT and salvage therapy for the M1 stage, the model established by baseline B-mode and SWE ultrasound combined with clinical and pathological indicators can predict the clinical response with better ability. Therefore, a more comprehensive ultrasound examination should be carried out before the intervention to provide critical information for clinicians to formulate personalized treatment strategies in the diagnosis stage.

## Data availability statement

The original contributions presented in the study are included in the article/**Supplementary Material**. Further inquiries can be directed to the corresponding author.

## Author contributions

Study conception and design: SW, WW and YP. Administrative support: YP and HZ. Provision of study materials or patient recruitment: XW, JL and HZ. Data collection: SW and ZL. Data analysis and interpretation: SW and WW. All authors contributed to the article and approved the submitted version.

## Supplementary material

Supplementary Table 1 Summary of the clinical and pathological indicators of the patients included in the test set. ER, estrogen receptor; PR, Progesterone receptor; HER2, human epidermal growth factor receptor-2; IHC subtype, immunohistochemistry subtype. *Quantitative data are mean ± standard deviation; P<0.05, the difference is statistically significant.

Supplementary Table 2 Summary of the B-mode US features of the patients included in the test set. BI-RADS, Breast imaging reporting and data system. *Quantitative data are mean ± standard deviation; Qualitative data are absent/present and percentage; P<0.05, the difference is statistically significant.

Supplementary Table 1 The distribution of the clinical and pathological indicators between test and validation sets. ER, estrogen receptor; PR, Progesterone receptor; HER2, human epidermal growth factor receptor-2; IHC subtype, immunohistochemistry subtype. *Quantitative data are mean ± standard deviation; P<0.05, the difference is statistically significant.

Supplementary Table 2 The distribution of the B-mode US features between test and validation sets. BI-RADS, Breast imaging reporting and data system. *Quantitative data are mean ± standard deviation; Qualitative data are absent/present and percentage; P<0.05, the difference is statistically significant.

Supplementary Table 3 The distribution of the SWE parameters between test and validation sets. *Data are mean ± standard deviation. P values for difference were determined by Mann-Whitney U test; P<0.05, the difference is statistically significant.
