# Supplementary material for: Prediction of clinical response to neoadjuvant therapy in advanced breast cancer by baseline B-mode ultrasound, shear-wave elastography, and pathological information
Source: Front Oncol. 2023 May 9;13:1096571. doi: 10.3389/fonc.2023.1096571 (PMC10203521; doi:10.3389/fonc.2023.1096571)
Supplement: Supplementary Table 1 — Summary of the clinical and pathological indicators of the patients included in the test set. ER, estrogen receptor; PR, Progesterone receptor; HER2, human epidermal growth factor receptor-2; IHC subtype, immunohistochemistry subtype. *Quantitative data are mean ± standard deviation; P<0.05, the difference is statistically significant. [file DataSheet_1.docx]

# Supplementary Material

Appendix TABLE A1 | Summary of the clinical and pathological indicators of the patients included in the test set.

| **Indicator** | **Total （n=152)** | **Non-responders（n=41）** | **Responders（n=111）** | ***P* Value** |
| --- | --- | --- | --- | --- |
| Age | - | 48.24±9.82 | 47.88±9.23 | 0.831 |
| Clinical T stage |  |  |  |  |
| 1 | 7(4.61) | 1(2.44) | 6(5.41) | 0.580 |
| 2 | 47(30.92) | 13(31.71) | 34(30.63) |  |
| 3 | 30(19.74) | 6(14.63) | 24(21.62) |  |
| 4 | 68(44.74) | 21(51.22) | 47(42.34) |  |
| Clinical N stage |  |  |  |  |
| 0 | 5(3.29) | 1(2.44) | 4(3.60) | 0.905 |
| 1 | 34(22.37) | 8(19.51) | 26(23.42) |  |
| 2 | 56(36.84) | 15(36.59) | 41(36.94) |  |
| 3 | 57(37.50) | 17(41.46) | 40(36.04) |  |
| Clinical M stage |  |  |  |  |
| 0 | 128(84.21) | 33(80.49) | 95(85.59) | 0.444 |
| 1 | 24(15.79) | 8(19.51) | 16(14.41) |  |
| Histologic type |  |  |  |  |
| Invasive ductal carcinoma | 124(81.58) | 35(85.37) | 89(80.18) | 0.464 |
| Others | 28(18.42) | 6(14.63) | 22(19.82) |  |
| ER% | - | 51.46±42.56 | 36.41±39.84 | 0.145 |
| Negative | 64(42.11) | 16(39.02) | 48(43.24) | 0.640 |
| Positive | 88(57.89) | 25(60.98) | 63(56.76) |  |
| PR% | - | 32.15±35.41 | 23.40±32.35 | 0.225 |
| Negative | 68(44.74) | 16(39.02) | 52(46.85) | 0.389 |
| Positive | 84(55.26) | 25(60.98) | 59(53.15) |  |
| HER2 | - | - | - | - |
| Negative | 89(58.55) | 34(82.93) | 55(49.55) | <0.001 |
| Positive | 63(41.45) | 7(17.07) | 56(50.45) |  |
| Ki-67% | - | 40.85±17.06 | 41.94±18.47 | 0.898 |
| <14% | 4(2.63) | 1(2.44) | 3(2.70) | 1.000 |
| ≥14% | 148(97.37) | 40(97.56) | 108(97.30) |  |
| IHC subtype |  |  |  |  |
| Luminal A | 4(2.63) | 1(2.44) | 3(2.70) | 0.420 |
| Luminal B | 84(55.26) | 24(58.53) | 60(54.05) |  |
| HER2+ | 31(20.39) | 5(12.20) | 26(23.42) |  |
| Triple negative | 33(21.71) | 11(26.83) | 22(19.82) |  |

ER, estrogen receptor; PR, Progesterone receptor; HER2, human epidermal growth factor receptor-2; IHC subtype, immunohistochemistry subtype.

* Quantitative data are mean ± standard deviation; P<0.05, the difference is statistically significant;

****Appendix TABLE A2 |** Summary of the B-mode US features of the patients included in the test set.**

| **Feature** | **Absent (-)/Present (+)** | **Total** | **Non-responders（n=41）** | **Responders（n=111）** | ***P* Value** |
| --- | --- | --- | --- | --- | --- |
| Size |  |  |  |  |  |
| dmax | / | / | 50.88±25.13 | 42.63±21.64 | 0.060 |
| Volume | / | / | 71956.78±112500.60 | 37894.73±57625.57 | 0.105 |
| Margin |  |  |  |  |  |
| Regular | + | 0(0.00) | 0(0.00) | 0(0.00) | / |
| Angular | - | 70(46.05) | 16(39.02) | 54(48.65) | 0.291 |
|  | + | 82(53.94) | 25(60.98) | 57(51.35) |  |
| Lobulated | - | 57(37.50) | 9(21.95) | 48(43.24) | 0.016 |
|  | + | 95(62.50) | 32(78.05) | 63(56.76) |  |
| Spiculated | - | 114(75.00) | 25(60.98) | 89(80.18) | 0.015 |
|  | + | 38(25.00) | 16(39.02) | 22(19.82) |  |
| Parallel | - | 14(9.21) | 2(4.88) | 12(10.81) | 0.262 |
|  | + | 138(90.79) | 39(95.12) | 99(89.19) |  |
| Calcifications | - | 18(11.84) | 6(14.63) | 12(10.81) | 0.517 |
|  | + | 134(88.16) | 35(85.37) | 99(89.19) |  |
| Posterior changes |  |  |  |  |  |
| Enhancement | - | 126(82.89) | 37(90.24) | 89(80.18) | 0.144 |
|  | + | 26(17.11) | 4(9.76) | 22(19.82) |  |
| Shadowing | - | 122(80.26) | 31(75.61) | 91(81.98) | 0.381 |
|  | + | 30(19.74) | 10(24.39) | 20(18.02) |  |
| Combined pattern | - | 99(65.13) | 23(56.10) | 76(68.47) | 0.155 |
|  | + | 53(34.87) | 18(43.90) | 35(31.53) |  |
| Peripheral tissue |  |  |  |  |  |
| Duct changes | - | 143(94.08) | 39(95.12) | 104(93.69) | 1.000 |
|  | + | 9(5.92) | 2(4.88) | 7(6.31) |  |
| Skin thickening | - | 114(75.00) | 26(63.41) | 88(79.28) | 0.045 |
|  | + | 38(25.00) | 15(36.59) | 23(20.72) |  |
| Skin edema | - | 129(84.87) | 31(75.61) | 98(88.29) | 0.053 |
|  | + | 23(15.13) | 10(24.39) | 13(11.71) |  |
| Invasion layers |  |  |  |  |  |
| Skin | - | 117(76.97) | 26(63.41) | 91(81.98) | 0.016 |
|  | + | 35(23.03) | 15(36.59) | 20(18.02) |  |
| Subcutaneous fat | - | 24(15.79) | 5(12.20) | 19(17.12) | 0.460 |
|  | + | 128(84.21) | 36(87.80) | 92(82.88) |  |
| Posterior mammary space | - | 24(15.79) | 2(4.88) | 22(19.82) | 0.025 |
|  | + | 128(84.21) | 39(95.12) | 89(80.18) |  |
| Muscle | - | 126(82.89) | 30(73.17) | 96(86.49) | 0.053 |
|  | + | 26(17.11) | 11(26.83) | 15(13.51) |  |
| Nipple | - | 145(95.39) | 39(95.12) | 106(95.50) | 1.000 |
|  | + | 7(4.61) | 2(4.88) | 5(4.50) |  |
| Lymph nodes | - | 6(3.95) | 0(0.0) | 6(5.41) | 0.294 |
|  | + | 146(96.05) | 41(100.0) | 105(94.59) |  |
| BI-RADS Classification |  |  |  |  |  |
| 4b | / | 3(1.97) | 1(2.44) | 2(1.80) | 0.034 |
| 4c | / | 19(12.50) | 1(2.44) | 18(16.22) |  |
| 5 | / | 130(85.53) | 39(95.12) | 91(81.98) |  |
| Adler grades |  |  |  |  |  |
| 0 | / | 15(9.87) | 4(9.76) | 11(9.91) | 0.802 |
| 1 | / | 52(34.21) | 13(31.71) | 39(35.14) |  |
| 2 | / | 36(23.68) | 12(29.27) | 24(21.62) |  |
| 3 | / | 49(32.24) | 12(29.27) | 37(33.33) |  |

BI-RADS, Breast imaging reporting and data system.

* Quantitative data are mean ± standard deviation; Qualitative data are absent/present and percentage; P<0.05, the difference is statistically significant.

Appendix TABLE B1 | The distribution of the clinical and pathological indicators between test and validation sets.

| **Indicator** | **Total (n=217)** | **Test set（n=152）** | **Validation set（n=65）** | ***P* Value** |
| --- | --- | --- | --- | --- |
| Age | 48.49±9.47 | 47.66±9.15 | 50.42±9.98 | 0.066 |
| Clinical T stage |  |  |  |  |
| 1 | 10(4.61) | 7(4.61) | 3(4.62) | 0.745 |
| 2 | 67(30.88) | 47(30.92) | 20(30.77) |  |
| 3 | 39(17.97) | 30(19.74) | 9(13.85) |  |
| 4 | 101(46.54) | 68(44.74) | 33(50.77) |  |
| Clinical N stage |  |  |  |  |
| 0 | 10(4.61) | 5(3.29) | 5(7.69) | 0.488 |
| 1 | 46(21.20) | 34(22.37) | 12(18.46) |  |
| 2 | 78(35.94) | 56(36.84) | 22(33.85) |  |
| 3 | 83(38.25) | 57(37.50) | 26(40.00) |  |
| Clinical M stage |  |  |  |  |
| 0 | 189(87.10) | 128(84.21) | 61(93.85) | 0.052 |
| 1 | 28(12.90) | 24(15.79) | 4(6.15) |  |
| Histologic type |  |  |  |  |
| Invasive ductal carcinoma | 183(84.33) | 124(81.58) | 59(90.77) | 0.088 |
| Others | 34(15.67) | 28(18.42) | 6(9.23) |  |
| ER % | 42.90±41.49 | 40.47±41.00 | 47.95±42.40 | 0.096 |
| Negative | 86(39.63) | 64(42.11) | 22(33.85) | 0.476 |
| Positive | 131(60.37) | 88(57.89) | 43(66.15) |  |
| PR % | 27.52±34.77 | 25.76±33.31 | 30.73±37.50 | 0.359 |
| Negative | 95(43.78) | 68(44.74) | 27(41.54) | 0.664 |
| Positive | 122(56.22) | 84(55.26) | 38(58.46) |  |
| HER2 |  |  |  |  |
| Negative | 131(60.37) | 89(58.55) | 42(64.62) | 0.403 |
| Positive | 86(39.63) | 63(41.45) | 23(35.38) |  |
| Ki-67 % | 40.90±18.42 | 41.64±18.05 | 39.53±19.18 | 0.307 |
| <14% | 6(2.76) | 4(2.63) | 2(3.08) | 1.000 |
| ≥14% | 211(97.24) | 148(97.37) | 63(96.92) |  |
| IHC subtype |  |  |  |  |
| Luminal A | 6(2.76) | 4(2.63) | 2(3.08) | 0.731 |
| Luminal B | 125(57.60) | 84(55.26) | 41(63.08) |  |
| HER2+ | 42(19.35) | 31(20.39) | 11(16.92) |  |
| Triple negative | 44(20.28) | 33(21.71) | 11(16.92) |  |

ER, estrogen receptor; PR, Progesterone receptor; HER2, human epidermal growth factor receptor-2; IHC subtype, immunohistochemistry subtype.

* Quantitative data are mean ± standard deviation; P<0.05, the difference is statistically significant.

Appendix TABLE B2 | The distribution of the B-mode US features between test and validation sets.

| **Feature** | **Absent (-)/Present (+)** | **Total （n=217)** | **Test set（n=152）** | **Validation set（n=65）** | ***P* Value** |
| --- | --- | --- | --- | --- | --- |
| Size |  |  |  |  |  |
| dmax | / | 44.51±23.16 | 44.86±22.84 | 43.20±23.89 | 0.618 |
| Volume | / | 55666.76±82977.81 | 57973.99±85453.05 | 47365.48±74190.14 | 0.349 |
| Margin |  |  |  |  |  |
| Regular | + | 0(0.00) | 0(0.00) | 0(0.00) | / |
| Angular | - | 98(45.16) | 70(46.05) | 28(43.08) | 0.687 |
|  | + | 119(58.84) | 82(53.94) | 37(56.92) |  |
| Lobulated | - | 77(35.48) | 57(37.50) | 20(30.77) | 0.343 |
|  | + | 140(64.52) | 95(62.50) | 45(69.23) |  |
| Spiculated | - | 167(76.96) | 114(75.00) | 53(81.54) | 0.295 |
|  | + | 50(23.04) | 38(25.00) | 12(18.46) |  |
| Parallel | - | 17(7.83) | 14(9.21) | 3(4.62) | 0.249 |
|  | + | 200(92.17) | 138(90.79) | 62(95.38) |  |
| Calcifications | - | 29(13.36) | 18(11.84) | 11(16.92) | 0.314 |
|  | + | 188(86.64) | 134(88.16) | 54(83.08) |  |
| Posterior changes |  |  |  |  |  |
| Enhancement | - | 180(82.95) | 126(82.89) | 54(83.08) | 0.974 |
|  | + | 37(17.05) | 26(17.11) | 11(16.92) |  |
| Shadowing | - | 170(78.34) | 122(80.26) | 48(73.85) | 0.293 |
|  | + | 47(21.66) | 30(19.74) | 17(26.15) |  |
| Combined pattern | - | 145(66.82) | 99(65.13) | 46(70.77) | 0.419 |
|  | + | 72(33.18) | 53(34.87) | 19(29.23) |  |
| Peripheral tissue |  |  |  |  |  |
| Duct changes | - | 206(94.93) | 143(94.08) | 63(96.92) | 0.512 |
|  | + | 11(5.07) | 9(5.92) | 2(3.08) |  |
| Skin thickening | - | 162(74.65) | 114(75.00) | 48(73.85) | 0.858 |
|  | + | 55(25.35) | 38(25.00) | 17(26.15) |  |
| Skin edema | - | 183(84.33) | 129(84.87) | 54(83.08) | 0.739 |
|  | + | 34(15.67) | 23(15.13) | 11(16.92) |  |
| Invasion layers |  |  |  |  |  |
| Skin | - | 167(76.96) | 117(76.97) | 50(76.92) | 0.994 |
|  | + | 50(23.04) | 35(23.03) | 15(23.08) |  |
| Subcutaneous fat | - | 37(17.05) | 24(15.79) | 13(20.00) | 0.450 |
|  | + | 180(82.95) | 128(84.21) | 52(80.00) |  |
| Posterior mammary space | - | 37(17.05) | 24(15.79) | 11(16.93) | 0.835 |
|  | + | 180(82.95) | 128(84.21) | 54(83.07) |  |
| Muscle | - | 184(84.79) | 126(82.89) | 55(84.62) | 0.755 |
|  | + | 33(15.21) | 26(17.11) | 10(15.38) |  |
| Nipple | - | 203(93.55) | 145(95.39) | 58(89.23) | 0.128 |
|  | + | 14(6.45) | 7(4.61) | 7(10.77) |  |
| Lymph nodes | - | 8(3.69) | 6(3.95) | 2(3.08) | 1.000 |
|  | + | 209(96.31) | 146(96.05) | 63(96.92) |  |
| BI-RADS Classification |  |  |  |  |  |
| 4b | / | 4(1.84) | 3(1.97) | 1(1.54) | 0.198 |
| 4c | / | 33(15.21) | 19(12.50) | 14(21.54) |  |
| 5 | / | 180(82.95) | 130(85.53) | 50(76.92) |  |
| Adler grades |  |  |  |  |  |
| 0 | / | 20(9.22) | 15(9.87) | 5(7.69) | 0.879 |
| 1 | / | 75(34.56) | 52(34.21) | 23(35.38) |  |
| 2 | / | 54(24.88) | 36(23.68) | 18(27.69) |  |
| 3 | / | 68(31.34) | 49(32.24) | 19(29.23) |  |

BI-RADS, Breast imaging reporting and data system.

* Quantitative data are mean ± standard deviation; Qualitative data are absent/present and percentage; P<0.05, the difference is statistically significant.

Appendix TABLE B3 | The distribution of the SWE parameters between test and validation sets

| **Parameters** | **Total (n=217)** | **Test set（n=152）** | **Validation set（n=65）** | ***P* Value** |
| --- | --- | --- | --- | --- |
| *Emax* | 8.84±1.70 | 8.95±1.68 | 8.58±1.74 | 0.074 |
| *Emin* | 7.57±2.04 | 7.69±2.02 | 7.27±2.08 | 0.148 |
| *Emedian* | 8.28±1.89 | 8.39±1.90 | 8.02±1.87 | 0.102 |
| *Emean* | 8.24±1.83 | 8.35±1.82 | 7.98±1.84 | 0.131 |
| Estd | 0.56±0.43 | 0.56±0.45 | 0.57±0.39 | 0.622 |

*Data are mean ± standard deviation. P values for difference were determined by Mann-Whitney U test; P<0.05, the difference is statistically significant.
